# Supplementary material for: Second-Generation Antiandrogen Therapy Radiosensitizes Prostate Cancer Regardless of Castration State through Inhibition of DNA Double Strand Break Repair
Source: Cancers (Basel). 2020 Aug 31;12(9):2467. doi: 10.3390/cancers12092467 (PMC7563746; doi:10.3390/cancers12092467)
Supplement: Supplementary file 1 [file cancers-12-02467-s001.pdf]

Supplementary Material: Second-Generation Antiandrogen Therapy Radiosensitizes Prostate Cancer Regardless of Castration State Through Inhibition of DNA Double Strand Break Repair

Mohamed E. Elsesy, Su Jung Oh-Hohenhorst, Anastassia Löser, Christoph Oing, Sally Mutiara, Sabrina Köcher, Stefanie Meien, Alexandra Zielinski, Susanne Burdak-Rothkamm, Derya Tilki, Hartwig Huland, Rudolf Schwarz, Cordula Petersen, Carsten Bokemeyer, Kai Rothkamm and Wael Y. Mansour

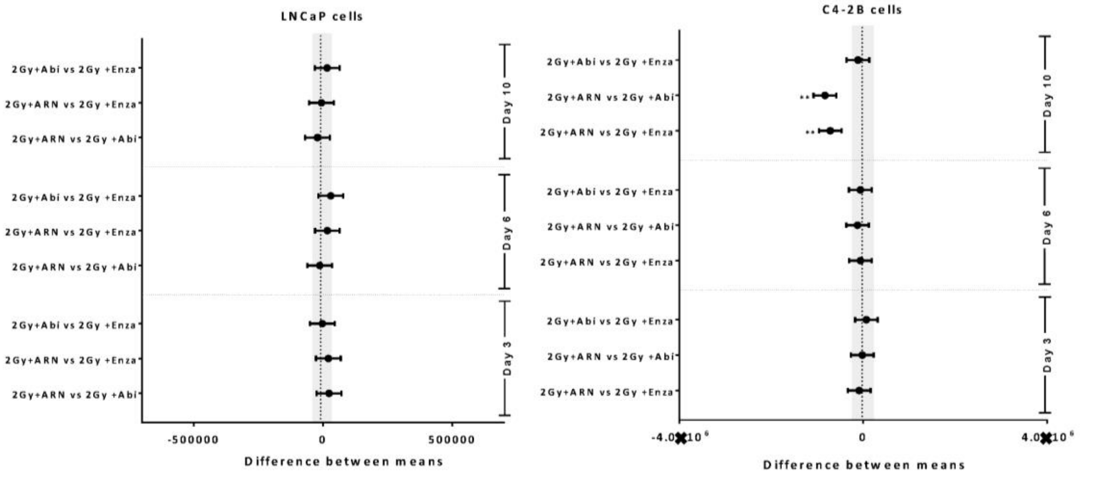

Figure S1. Multiple comparisons between the combination of the indicated antiandrogens and IR in (**A**) LNCaP and (**B**) C4-2B cells. Tukey's multiple comparisons test was used to compare between the indicated treatments pairs in LNCaP or C4-2B cells. Significance was measured using two way ANOVA test. Significance is indicated as * for the $p < 0.05$, ** for $p < 0.001$ and *** for $p < 0.0001$. ns: not significant.

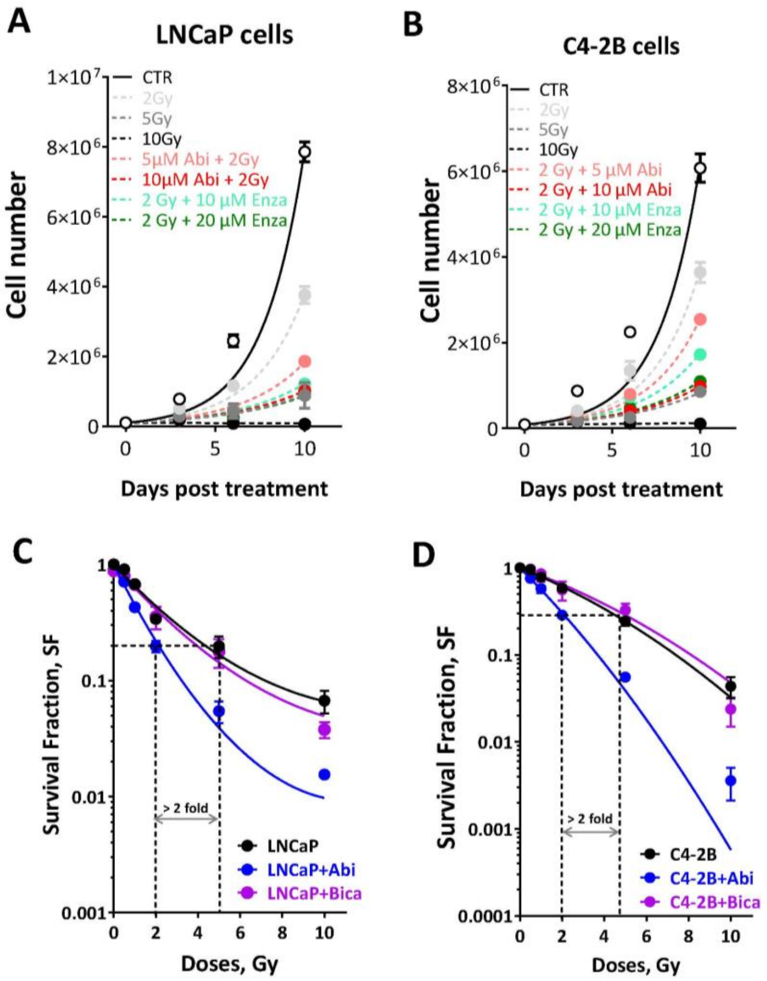

Figure S2. Second generation ADT therapy enhances the IR effect at least 2 fold. Cell numbers were determined in LNCaP (**A**) or C4-2B cells (**B**) on days 0, 3, 6 and 10 post treatment with the indicated second generation antiandrogens. (**C**,**D**) Survival fractions were measured using agarose CFA for LNCaP (C) and C4-2B (D) cells treated with 5 μM abiraterone acetate or 10 μM bicalutamide before irradiation with the indicated X-ray does. Shown are means ±SEM of at least three independent experiments.

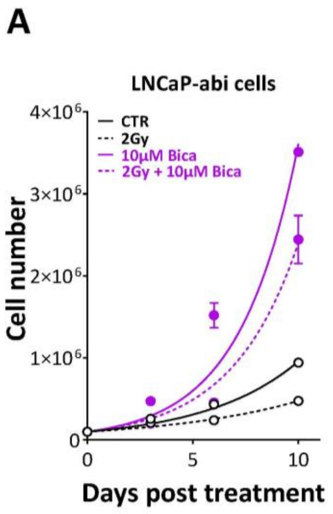

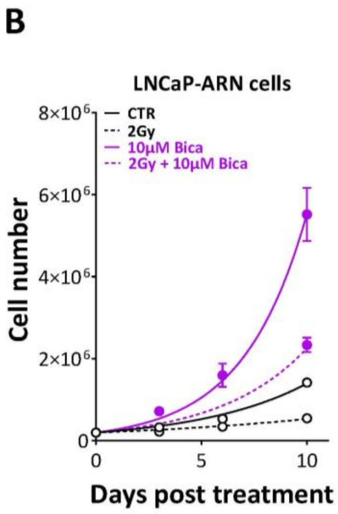

Doubling time, days (A)

CTR	2Gy	10µM Bica	10µM Bica +2Gy
3.069	1.933	4.444	2.180

Doubling time, days (B)

CTR	2Gy	10µM Bica	10µM Bica +2Gy
3.565	2.086	7.051	2.860

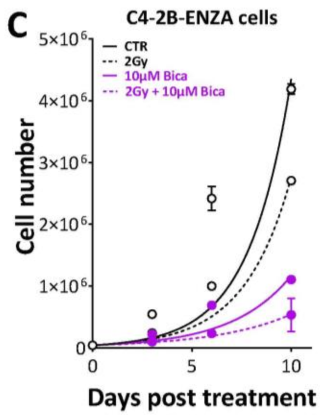

Doubling time, days

CTR	2Gy	10µM Bica	10µM Bica +2Gy
1.478	2.314	1.637	2.658

Figure S3 Bicalutamide showed contradictory effects on the cytotoxicity of ionizing radiation in hormone resistant cells. Cell number was determined in LNCaP-abi (**A**), LNCaP-ARN (**B**) or C4-2BENZA cells (**C**) on days 0, 3, 6 and 10 post treatment with 10 µM bicalutamide (Bica). Cell doubling time in days was calculated for each treatment by fitting exponential growth curves using GraphPad Prism 7. Shown are means ±SEM of at least three independent experiments.

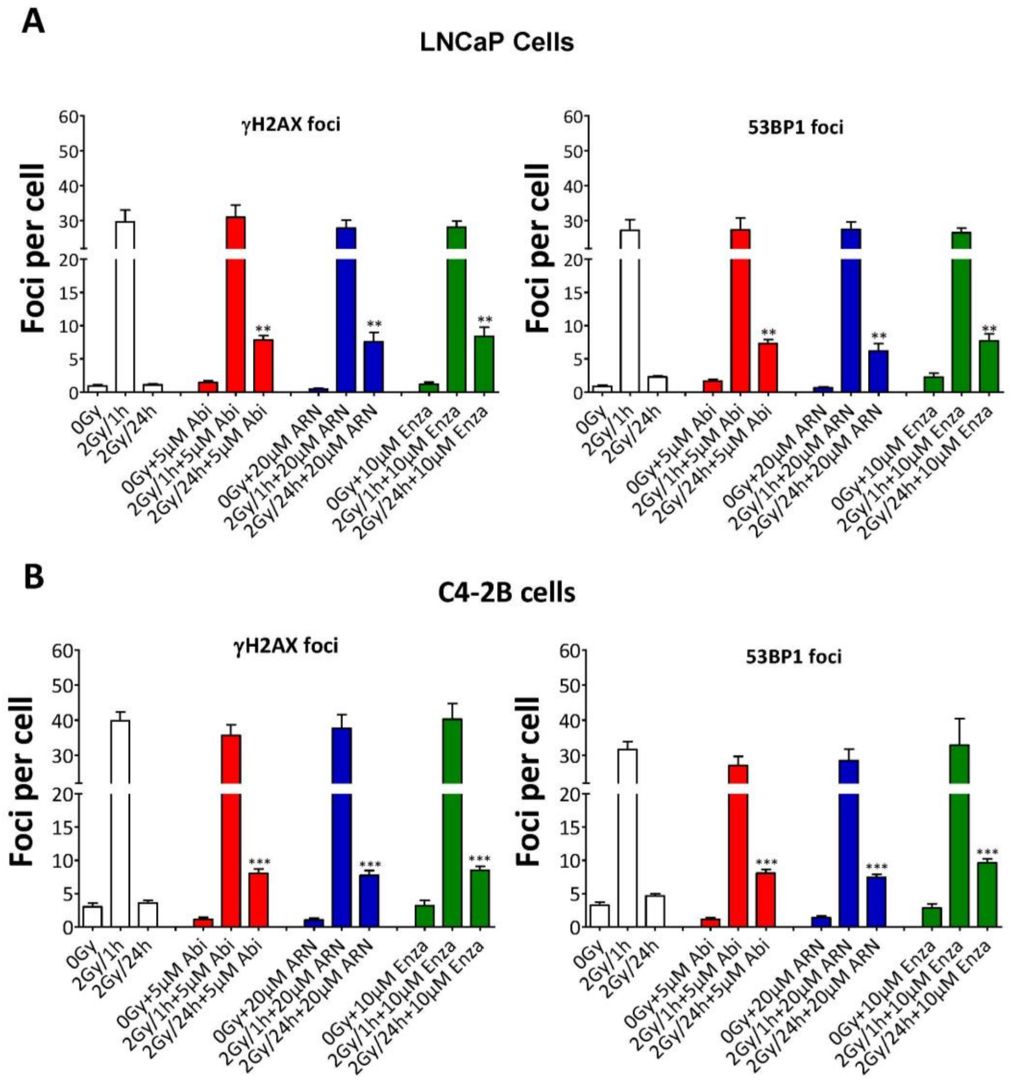

Figure S4. Quantitation of individual yH2AX (left panel) and 53BP1 (right panel) foci of the experiments performed in Figure 7. in (**A**) LNCaP and (**B**) C4-2B cells. At least 100 cells were analyzed. Shown are the means ±SEM from at least three independent experiments. *p*-values were calculated using the Mann-Whitney U test. Significance is indicated as * for the $p < 0.05$, ** for $p < 0.001$ and *** for $p < 0.0001$. ns: not significant.

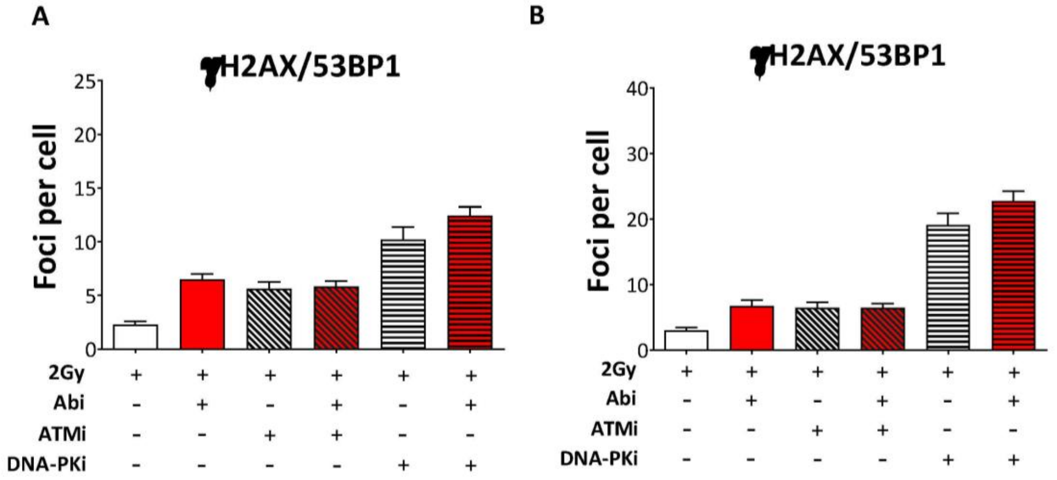

Figure S5. Quantitation of ◎H2AX/53BP1 foci at 24h post 2Gy in (**A**) LNCaP and (**B**) C4-2B cells after treatment with 2.5 μM ATM or 5 μM DNA-PKcs inhibitor (2 h pre-IR) and 5 μM abiraterone acetate (24 h-pre-IR) either individually or combined. At least 100 cells were analyzed. Shown are the means ±SEM from at least three independent experiments.

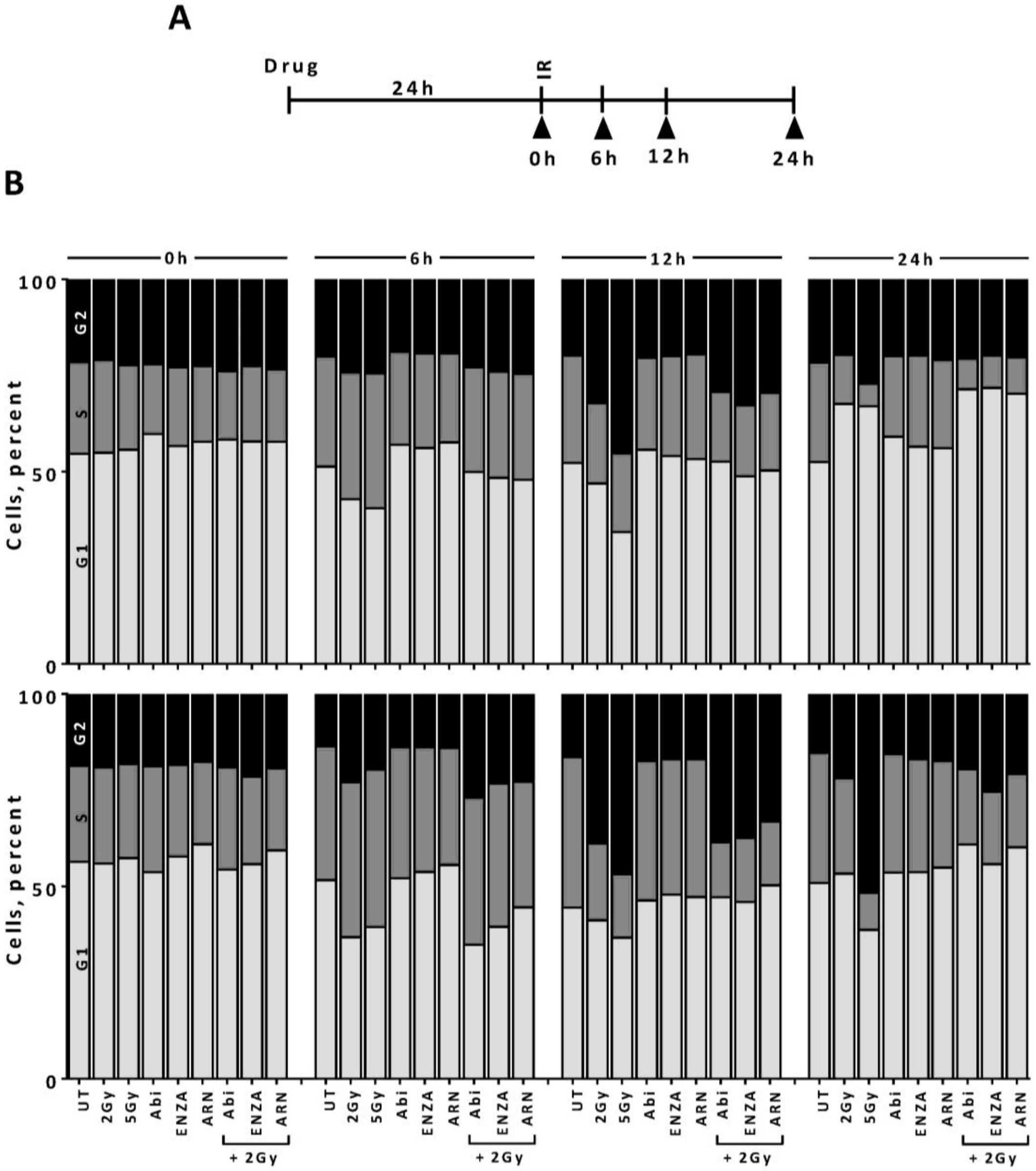

Figure S6. Second generation AHT does not affect cell cycle distribution after IR. (**A**) Schematic representation for the experiment flow. cells were treated with the indicated AHT for 24 hours before being irradiated with 2Gy and cell cycle profiles of LNCaP (upper panel) and C4-2B (lower panel) were determined by propidium iodide staining and flow cytometry at the indicated time points. (**B**) The percentage of cells from each treatment in G1 (light grey), S (dark grey) or G2 (black) phase are shown.

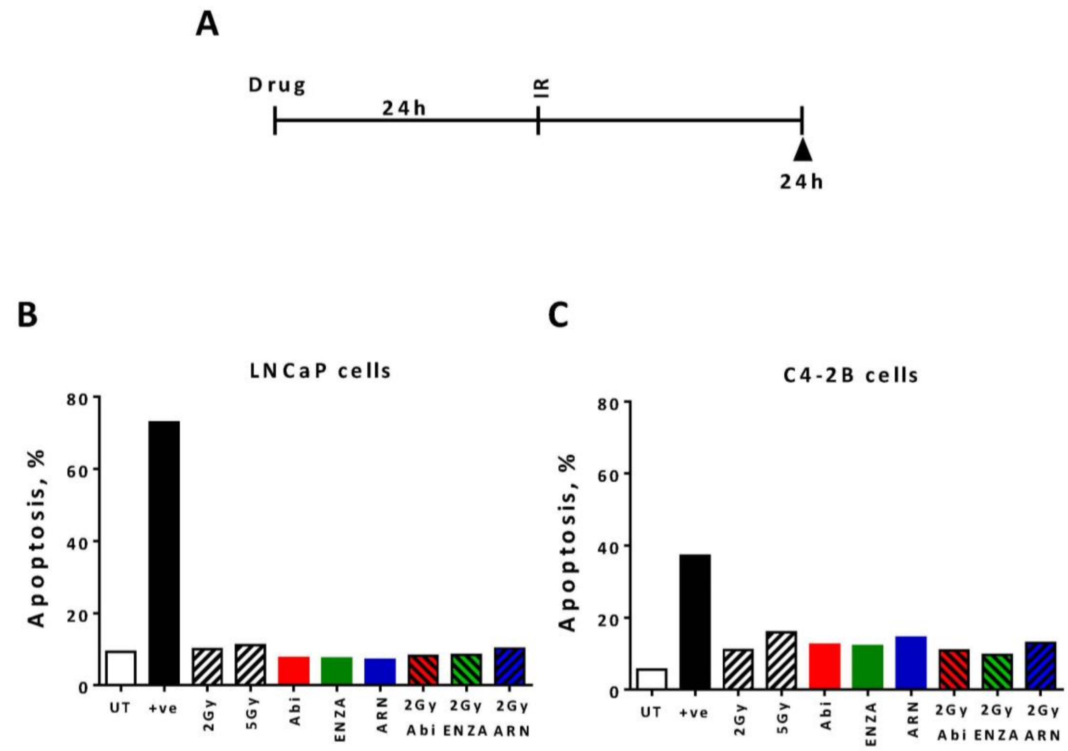

Figure S7. Second generation AHT does not increase apoptosis after IR. (**A**) Schematic representation for the experiment flow. Both LNCaP (**B**) and C4-2B (**C**) cells were treated the indicated antiandrogen for 24 hours before being irradiated with 2Gy and apoptosis were determined via caspase activity. As a positive control (+ve) for apoptosis induction, cells were treated with 1 μM staurosporine for 12 h.

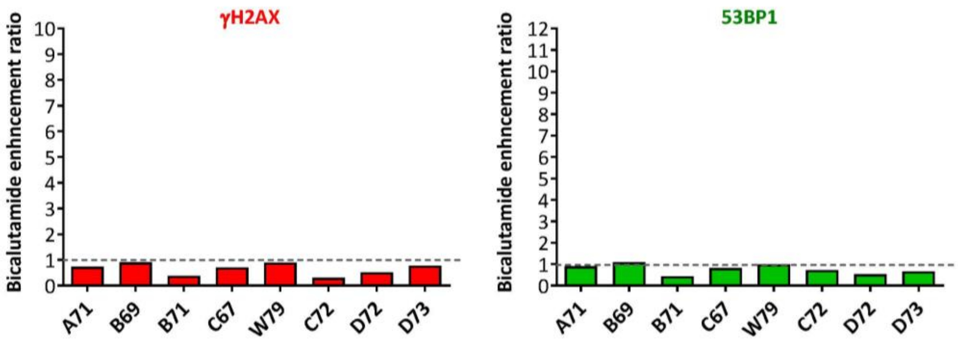

Figure S8. Bicalutamide does not inhibit DSB repair after IR. Bicalutamid-mediated enhancement ratio (BicaER) on gH2AX (left panel) and 53BP1 (right panel) foci of 8 punch biopsies from 8 PCa patients.

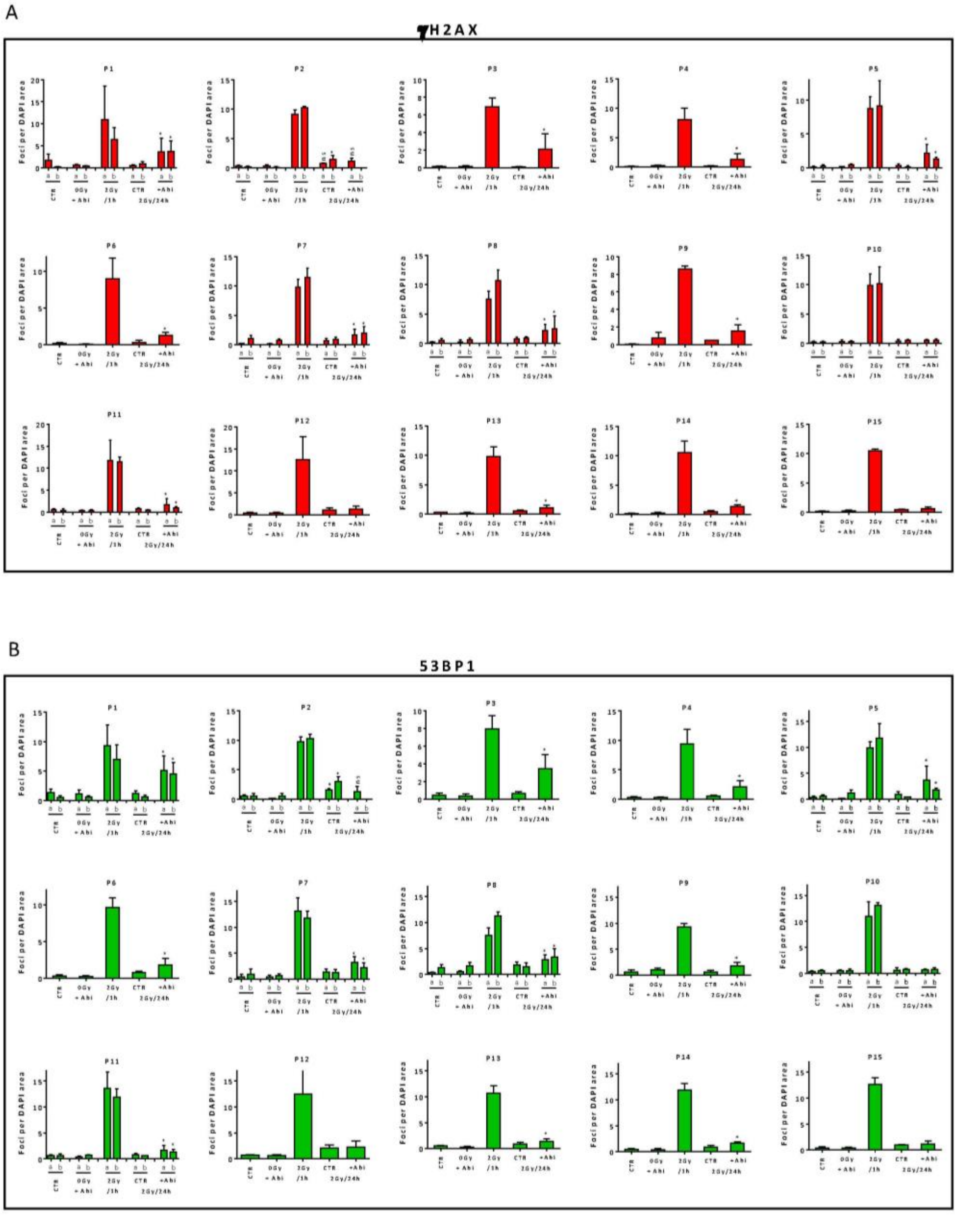

Figure S9. Abiraterone acetate efficiently suppresses DSB-repair in fresh PCa tissues after ex-vivo irradiation. Number of (**A**) yH2AX or (**B**) 53BP1 foci in 22 punch biopsies from the 15 PCa patients after the indicated treatments. Shown are the means ±SEM from at least three independent experiments. *p*-values were calculated using the Mann-Whitney U test. Significance is indicated as * for the $p < 0.05$, ** for $p < 0.001$ and *** for $p < 0.0001$. ns: not significant.

© 2020 by the authors. Licensee MDPI, Basel, Switzerland. This article is an open access article distributed under the terms and conditions of the Creative Commons Attribution (CC BY) license (http://creativecommons.org/licenses/by/4.0/).
